# Oral Health, Social and Emotional Well-Being, and Economic Costs: Protocol for the Second Australian National Child Oral Health Survey

**DOI:** 10.2196/52233

**Published:** 2023-11-14

**Authors:** Lisa Jamieson, Liana Luzzi, Sergio Chrisopoulos, Rachel Roberts, Peter Arrow, Sanjeewa Kularatna, Murthy Mittinty, Dandara Haag, Pedro Henrique Ribeiro Santiago, Gloria Mejia

**Affiliations:** 1 Australian Research Centre for Population Oral Health Adelaide Dental School The University of Adelaide Adelaide Australia

**Keywords:** Australian children, cost-effective analysis, dental caries, social and emotional well-being

## Abstract

**Background:**

Given the significant investment of governments and families into the provision of child dental care services in Australia, continued population oral health surveillance through national oral health surveys is imperative.

**Objective:**

The aims of this study are to conduct a second National Child Oral Health Survey (NCOHS-2) to (1) describe the prevalence, extent, and impact of oral diseases in contemporary Australian children; (2) evaluate changes in the prevalence and extent of oral diseases in the Australian child population and socioeconomic subgroups since the first National Child Oral Health Study (NCOHS-1) in 2012-2013; and (3) use economic modeling to evaluate the burden of child oral disease from the NCOHS-1 and NCOHS-2 and to estimate the cost-effectiveness of targeted programs for high-risk child groups.

**Methods:**

The NCOHS-2 will closely mimic the NCOHS-1 in being a cross-sectional survey of a representative sample of Australian children aged 5-14 years. The survey will comprise oral epidemiological examinations and questionnaires to elucidate associations between dental disease in a range of outcomes, including social and emotional well-being. The information will be analyzed within the context of dental service organization and delivery at national and jurisdictional levels. Information from the NCOHS-1 and NCOHS-2 will be used to simulate oral disease and its economic burden using both health system and household costs of childhood oral health disease.

**Results:**

Participant recruitment for the NCOHS-2 will commence in February 2024. The first results are expected to be submitted for publication 6 months after NCOHS-2 data collection has been completed. Thematic workshops with key partners and stakeholders will also occur at this time.

**Conclusions:**

Regular surveillance of child oral health at an Australian level facilitates timely policy and planning of each state and territory’s dental public health sector. This is imperative to enable the most equitable distribution of scarce public monies, especially for socially disadvantaged children who bear the greatest dental disease burden. The last NCOHS was conducted in 2012-2014, meaning that these data need to be updated to better inform effective dental health policy and planning. The NCOHS-2 will enable more up-to-date estimates of dental disease prevalence and severity among Australian children, with cost-effective analysis being useful to determine the economic burden of poor child dental health on social and emotional well-being and other health indicators.

**International Registered Report Identifier (IRRID):**

PRR1-10.2196/52233

## Introduction

### Overview

Dental caries is the most common childhood condition in Australia, resulting in costly treatment (up to Aus $ 1 billion [US$ 651,190,049] dollars annually), poor school performance, inadequate nutrition, problems with sleeping, and adverse mental health (social and emotional well-being) [1]. In addition to treatment costs, there are productivity losses due to caregiver absenteeism from work [2]. The burden of child oral diseases is overrepresented among socially vulnerable groups. For example, in the first National Child Oral Health Study (NCOHS-1) from 2012-2014, a total of 23% of children aged 5-10 years had dental caries in the primary dentition compared to 36% of Indigenous children, and 41% of non-Indigenous children aged 6-14 years had dental caries in the permanent dentition compared to 61% of Indigenous children [3]. From 2016-2017, approximately 70,200 hospitalizations for dental conditions could have been prevented; these hospitalizations were highest among socially vulnerable children [4].

As described in Australia’s National Oral Health Plan 2015-2024 [5], there is a lack of routinely collected population-based dental data in Australia. This affects the capacity to effectively evaluate the impact of local and national policies, programs, and service models, which, in turn, makes it difficult to translate research and evaluation findings into policy and improved models of care. The NCOHS-1 has been influential in the development of public policy in Australia. It has provided valuable information on the oral health outcomes of children from different socioeconomic backgrounds and identified a number of underlying causes of oral health inequities, thus providing a sound evidence base for selecting interventions to reduce these inequalities.

### Impact of Child Dental Disease on Social and Emotional Well-Being and Economic Productivity

Evidence shows that chronic illness is a major contributor to school absenteeism in childhood, thus impacting child school performance and social or emotional well-being [6]. Guarnizo-Herreno and Wehby [7] assessed associations between poor dental health and school performance or psychosocial well-being in a nationally representative sample of over 40,000 children in the United States. They reported that children with dental caries were more likely to have problems at school (odds ratio [OR] 1.52, 95% CI 1.37-1.72), more likely to miss school (OR 1.42, 95% CI 1.23-1.64), and less likely to do all required homework (OR 0.76, 95% CI 0.68-0.85). Dental problems among schoolchildren were associated with shyness, unhappiness, feelings of worthlessness, and a reduced ability to make friends. This may have far-reaching consequences in adulthood, affecting both economic productivity and meaningful engagement in civic activities. To the best of our knowledge, there has been no analysis of the association between poor dental health and Australian children’s social and emotional well-being; this is an important strategic priority with the Australian Government launching the world’s first National Children’s Mental Health and Wellbeing Strategy in October 2021 [8]. Poor oral health in childhood also has an immediate impact on caregiver workplace productivity, with some dental procedures for children requiring care under a hospital-based general anesthetic. These services are only available in major metropolitan locations and typically have long waitlists [9]. This means caregivers need to take substantial leave from employment, with out-of-pocket costs including transport, accommodation, and childcare arrangements [10].

### Provision of Dental Care for Australian Children

The provision of dental services for children varies by jurisdiction in Australia, although the federally operated (through Medicare) Child Dental Benefits Schedule (CDBS) is available for families already receiving other benefits, such as the Family Tax Benefit [11]. This scheme provides each eligible child up to Aus $1052 (US $ 668.96) over 2 calendar years for covering checkups, routine cleaning, simple restorations, extractions, and root canals. It does not cover orthodontic treatment, hospital-based dental care, or cosmetic dental procedures. Children who are eligible for the CDBS can receive treatment in any public dental clinic, with the claim being bulk billed with no out-of-pocket costs. Waitlists in public dental services, up to 2 years in some jurisdictions, are common. Some, but not all, private dentists also accept CDBS payments. Children not eligible for the CDBS need to pay for dental care out-of-pocket, or through dental insurance, in the private sector. In 2019, the Australian Government paid CDBS benefits of US $324,483,573 for 5,450,996 dental services across Australia, averaging US $59.53 in benefits per service. The average benefits paid per service ranged from US $40 for a diagnostic service to US $138 for a restorative service [12]. Data from the 2014-2015 National Health Survey indicated that around 1 in 5 children aged 2-14 years had never visited a dental professional [13]. Estimates from the 2013 National Dental Telephone Interview Survey showed that around 1 in 5 children aged 5-14 years had last visited a school dental service and around 2 in 3 had last visited a private dental practice. The proportion of children aged 5-14 years who had last visited a school dental service more than halved over the 1994-2013 period, from 54% to 21% [14].

### Dental-Related Child Hospitalizations

Reducing rates of hospital-based child dental service provision is a key performance indicator of the 2015-2024 National Oral Health Plan [5]. The rate of preventable hospitalizations for dental conditions is influenced by the adequacy of preventive and primary dental care services, the prevalence of severe dental disease in the community, and the availability and accessibility of appropriate community and hospital-based dental services [5]. From 2017-2018, the rate of preventable hospitalizations due to dental conditions was highest among those aged 5-9 years (9.5 per 1000 population). Some children receive dental care under general anesthesia, usually due to the severity of the disease or other medical, physical, or behavioral complications. Dental care under general anesthesia is resource intensive and not without risk.

### Calculating the Economic Impact of Child Dental Disease

In Australia, it has been estimated that over 10,000 days of work and 2.5 million school hours are lost each year because of dental-related illnesses [15]. Identifying the economic burden of child dental disease is useful to understand the maximum number of resources that could be saved or gained if that disease were partially or fully eradicated. While there has been some attempt to quantify the indirect costs to government and society of child dental disease on the health system, there is a lack of consistent quantitative analysis and information. Robust data and economic analysis are urgently required to quantify these costs and the indirect financial pressures that child dental disease places on government and society. While treatment is a direct and costly consequence of child oral disease, reductions in morbidity may also imply other economic benefits. For example, indirect costs include productivity losses due to absenteeism from school and work, yet relatively little evidence exists in this regard.

### Child Oral Health Surveys in Australia

The 2012-2014 NCOHS-1 was a cross-sectional study of the child population aged 5-14 years in Australia [3]. A total of 24,664 children from 841 participating schools were involved. Children were selected using a complex, multistage, and stratified sampling design, with a sophisticated weighting procedure implemented to adjust for variations in probabilities of selection and response rates. Information was collected through a parental questionnaire and a detailed dental examination by trained dental professionals. The findings suggested substantial social patterning of oral health status, dental service use, and dental and general health behaviors among Australian children. It was clear that addressing factors at the child, family, school, and community levels was necessary for reducing social inequalities in child oral health.

The proposed research will build upon the already established population-based epidemiological platform of the NCOHS-1, with the conduct of a second National Child Oral Health Survey (NCOHS-2), enabling a follow-up snapshot of a large and representative national child cohort. The specific study aims to (1) describe the prevalence and extent of oral diseases and their associations with social and emotional well-being and other outcomes in contemporary Australian children, (2) evaluate changes in the prevalence and extent of oral diseases in the Australian child population and socioeconomic subgroups since the NCOHS-1, and (3) use economic modeling to evaluate the burden of child oral disease from the NCOHS-1 and NCOHS-2 and to estimate the efficacy and cost-effectiveness of targeted programs for high-risk child groups.

## Methods

The NCOHS-2 will be the second representative child oral health survey conducted in Australia in partnership with the dental public health sector of each of Australia’s states and territories and the Federal Department of Health.

### Study Design

The study design will closely mimic the NCOHS-1 in being a cross-sectional survey of a representative sample of Australian children aged 5-14 years. The survey will comprise oral epidemiological examinations and questionnaires to elucidate the impacts of dental disease on social and emotional well-being and other outcomes. The information will be analyzed within the context of dental service organization and delivery at national and jurisdictional levels. A key goal is to evaluate changes in the prevalence and extent of oral diseases in the Australian child population and socioeconomic subgroups since the NCOHS-1, using age-period-cohort analysis (NCOHS-1 and NCOHS-2, with both cohorts aged 5-14 years).

### Sampling and Recruitment

As with the NCOHS-1, in the NCOHS-2, a multistage, stratified, and clustered random sampling design will be implemented to ensure sufficient geographic representation from both urban and rural settings in all states and territories while optimizing the efficiency of fieldwork for dental examiners. The sampling frame will include public and private schools in all states and territories. As with the NCOHS-1, NCOHS-2 participants will be selected in each state or territory using a 2-stage stratified clustered sampling design with a combined allocation of children per state proportional to population size.

### Data Collection

#### Oral Epidemiological Examination

All selected children will be invited to undergo a standardized oral epidemiological examination at school-based units organized by state- or territory-appointed dental professionals. Small teams of examiners and data recorders (3-10 examining teams per state or territory) will be trained in the clinical assessment of key oral epidemiological outcomes. All NCOHS-2 examiners will be calibrated and tested in the field against a gold standard examiner to estimate interexaminer reliability and to ensure rigor and consistency of measurement. To facilitate comparisons across time, the oral outcome measures in the NCOHS-2 will be the same as those assessed in the NCOHS-1 using accepted indices and benchmark measures for large oral epidemiological surveys. The outcomes will include the number of teeth, experience of dental caries, dental fluorosis, molar incisor hypomineralization, dental plaque, and dental trauma.

#### Self-Report Questionnaire

The caregiver-reported questionnaire in the NCOHS-2 will comprise items on sociodemographics, self-rated oral health, self-rated general health, items used for cost-effective analysis (Dental Caries Utility Index) [16], oral health-related quality of life (Child Perceptions Questionnaire 11-14) [17] and Caries Impacts and Experiences Questionnaire for Children quality of life [18]), dental service use, dental behaviors, psychosocial factors, and social and emotional well-being. Social and emotional well-being will be assessed using the Stirling Children’s Wellbeing Scale, a 15-item instrument developed by the Stirling Educational Psychology Service to measure emotional and psychological well-being among children aged 8-15 years [19]. The Stirling Children’s Wellbeing Scale has been validated and displayed good psychometric properties across multiple countries, including Scotland [19], Japan [20], and Bangladesh [21], among others [22].

### Sample Size

The minimum required sample size was determined using standard methods to address the specific aims of the NCOHS-2 [23]. The main outcomes evaluated were the “decayed, missing, and filled teeth” (DMFT) index, the “decayed, missing, and filled surfaces” index, the percentage of children with untreated decay, and the percentage of children with overall caries experience. These outcomes were evaluated for primary dentition, among the age groups of 5-6, 7-8, and 9-10 years, and permanent dentition, among the age groups of 6-8, 9-11, and 12-14 years. The sample size was calculated to detect a 40% difference in the mean of the outcome or a 40% difference in the prevalence of the outcome with a power of 80% and a significance level of 5%. Sample size requirements were calculated for a simple random sample and then adjusted by the design effect to account for the NCOHS-2–stratified, 2-stage sampling design [24]. The design effect values were estimated based on existing NCOHS 2012-2014 data, which included information on children’s oral health status (for all abovementioned outcomes) [3] and were used as a proxy in the calculation formula to estimate sample sizes for the NCOHS-2 [25]. The estimated sample size analysis was conducted with R software [26] and the R packages *survey* [27] and *pwr* [28]. The minimally required overall sample size of the NCOHS-2 was based on the outcome that required the largest sample—the percentage of children with untreated decay in the permanent dentition (n=16,678)—meaning that the NCOHS-2 would be adequately powered to detect all other outcomes. Because the percentage of children with untreated decay in the permanent dentition was not evaluated for children aged 5 years, an additional sample size (n=885)—estimated based on the sample required to evaluate the percentage of children with untreated decay in the primary dentition at age 5 years—was added to constitute the NCOHS-2 minimally required overall sample size (n=17,563). The sample allocation per state was conducted using combined allocation, consisting of an unweighted sum of equal allocation and proportional allocation [29]. The state and territory sample sizes were then further upwardly increased to meet jurisdiction objectives (N=18,700; Northern Territory, n=1400; Australian Capital Territory, n=1400; Tasmania, n=1400; South Australia, n=1850; Western Australia, n=2150; Queensland, n=3000; Victoria, n=3500; Norfolk Island, n=4000).

### Economic Modeling

Information from the NCOHS-1 and NCOHS-2 will be used to simulate oral disease and its economic burden using both health system and household costs of childhood oral health disease. The cost of illness approach will be used to estimate the economic burden of dental disease. Direct and indirect costs will be estimated using available and collected information. Direct health system costs will include dental, pharmaceutical, emergency department, hospitalization, specialist appointments, diagnostic testing or pathology, and other health care services related to the provision of dental treatment. Indirect patient costs (out-of-pocket) will include Medicare Benefits Schedule and Pharmaceutical Benefits Scheme item copayments and other out-of-pocket costs, income loss for parents and children after attaining adulthood, and travel costs. Indirect societal financial costs will include other direct health system costs, patient financial costs, business financial costs, and government costs.

### Statistical Analysis

We will specifically test for each aim.

- Aim 1: to evaluate the prevalence and extent of oral diseases and their association with social and emotional well-being and other outcomes in contemporary Australian children
- Aim 2: to evaluate changes in Australian child dental disease

The design of 2 cross-sectional, nationally representative surveys will enable comparisons between 2 time points from the NCOHS-1 and NCOHS-2 to be conducted. The main outcomes will be the prevalence and extent of decayed, missing, and filled teeth in the primary and permanent dentition (percentage of DMFT>0 and mean DMFT). Analysis will be performed progressively from descriptive to explanatory multivariable analysis, multilevel regression models, and causal inference models, accounting for systematic differences between the 2 periods, including family and area socioeconomic status, model of dental service provision, exposure to fluoride, dental behaviors, and diet. SAS (SAS Institute) or SUDAAN (RTI International), STATA (StataCorp), and R software will be used to generate sample estimates and standard errors and adjust for the complex sampling design of each survey.

- Aim 3: to evaluate the child oral disease burden from the NCOHS-1 and NCOHS-2 and estimate the efficacy and cost-effectiveness of targeted programs for high-risk child groups

Economic modeling using the R and TreeAge software (TreeAge Software, LLC) will be used to assess variations in cost-effectiveness according to accessibility and cost of child dental care. Different cutoffs and thresholds will be used to determine the most cost-effective models for targeted child oral health programs, preventive dental services, and insurance plans for optimal economic productivity. The analysis will involve the estimating costs incurred by the child population and population subgroups. Other costs will be estimated from a societal perspective, including productivity effects (based on expected time off work associated with child dental conditions and treatment) and dental service costs incurred by insurance companies and taxpayers. In collaboration with the Australian Institute of Health and Welfare’s Australian Burden of Disease Unit, NCOHS-1 and NCOHS-2 estimates will be used to project the burden of child oral diseases in the 2020 decade and beyond. Markov models will use both health system and societal perspectives according to the cost being considered. The economic analysis will use cost-effectiveness (natural units as outcome measures) and cost utility analysis (quality-adjusted life years as outcome measures) depending on the outcome measure. Deterministic and probabilistic sensitivity analyses will be performed for all model base-case analyses.

### Ethical Considerations

Ethics approval has been obtained from the University of Adelaide Human Research Ethics Committee (37659 H-2023). Before being recruited, all participants will be required to sign an informed consent form, which includes consent for the authors to publish the findings in peer-reviewed scientific literature. Data will be de-identified and there will be no compensation provided. All authors are named investigators on the project; they all contributed to the intellectual input of the study design and in writing this protocol.

## Results

Participant recruitment for the NCOHS-2 will commence in February 2024. The first results are expected to be submitted for publication 6 months after NCOHS-2 data collection has been completed. Thematic workshops with key partners and stakeholders will also occur at this time.

## Discussion

### Overview

Regular surveillance of child oral health at an Australian level facilitates timely policy and planning in each state’s and territory’s dental public health sector. This is imperative to enable the most equitable distribution of scarce public monies, especially for socially disadvantaged children who bear the greatest dental disease burden. The last NCOHS was conducted in 2012-2014, with these data being effectively outdated in terms of contemporary and effective dental health policy and planning [30]. The NCOHS-2 will enable more up-to-date estimates of dental disease prevalence and severity among Australian children, with cost-effective analysis being useful to determine the economic burden over a lifetime of poor child dental health on social and emotional well-being and other health indicators.

As described in the National Oral Health Plan 2015-2024, there is a lack of comprehensive, routinely collected population-based dental data in Australia. This affects the capacity to effectively evaluate the impact of local and national policies, programs, and service models, which, in turn, makes it difficult to translate research and evaluation findings into policy and improved models of care. Both the National Oral Health Plan 2015-2024 and the previous National Oral Health Plan 2004-2013 call for regular national data collections of oral health to ensure quality planning, monitoring, and evaluation at both population and service levels. The previous NCOHS has been influential in the development of public policy in Australia. It has provided valuable information on the oral health outcomes of children from different socioeconomic backgrounds and allowed the diagnosis of a number of underlying causes of inequalities in oral health, thus providing a sound evidence base for selecting interventions to reduce these inequalities. It is expected that the results derived from this project will be important in informing public policy at both the state and national level over the next decade.

Optimal prevention and treatment of child dental disease, which in turn will improve child social and emotional well-being and economic productivity in the long term, requires appropriate health care systems, organizational governance, infrastructure, and resources that are evidence-based.

### Outcomes and Significance

The National Children’s Mental Health and Wellbeing Strategy, launched by the Australian Government in 2021, identifies the importance of improving Australian child health and well-being [8]. Evidence shows that dental problems among schoolchildren are associated with shyness, unhappiness, feelings of worthlessness, and a reduced ability to make friends. Linking the impact of poor dental health on Australian child school and social and emotional well-being using a large, nationally representative data set will lead to crucial insights regarding this prevalent and preventable childhood condition. Using cutting-edge cost-effectiveness modeling will be a significant contribution to dental service policy and planning to estimate the projected burden of child oral disease from 2012 and beyond, the impact on economic productivity over the life course, and the efficacy and cost-effectiveness of targeted programs for high-risk child groups. With strong partnerships with key state and territory and Commonwealth stakeholders, the findings of the NCOHS-2 will enable complex questions to be answered that will guide policy on improving child oral health that is relevant for all Australian children.

## Appendix

Multimedia Appendix 1 Peer-review report by the Australian Government National Health and Medical Research Council.

## Abbreviations

CDBS: Child Dental Benefits Schedule
DMFT: decayed, missing, and filled teeth
NCOHS-1: first National Child Oral Health Study
NCOHS-2: second National Child Oral Health Survey
OR: odds ratio

## Data Availability

The data sets generated during this study are not publicly available due to privacy issues of the participants but are available from the corresponding author on reasonable request.
