# Supplementary material for: Oral Health, Social and Emotional Well-Being, and Economic Costs: Protocol for the Second Australian National Child Oral Health Survey
Source: JMIR Res Protoc. 2023 Nov 14;12:e52233. doi: 10.2196/52233 (PMC10691528; doi:10.2196/52233)
Supplement: Multimedia Appendix 1 [file resprot_v12i1e52233_app1.pdf]

Australian Government
National Health and Medical Research Council

NHMRC

Application Assessment Summary

2022 Partnership Projects PRC1

Application ID: 2016736

Chief Investigator A: Professor Lisa Jamieson

Administering Institution: The University of Adelaide

Table 1: Summary of the assessment of your application against the **2022 Partnership Projects PRC1** Grant Assessment Criteria.

Criteria			
	1	1. Track records of the Chief Investigators, Partner Organisations and Partner Investigators, relative to opportunity (25%)	5.222
	2	2. Scientific quality of the proposal and methodology (25%)	4.444
	3	3. Relevance and likelihood to influence health policy and practice (25%)	5.000
	4	4. Strength of the partnership (25%)	5.333
		**Overall Score**	**5.000**
		**Category**	**5**

Table 2: The proportion of Partnership Project applications in each category.

The table provides a summary of all Partnership Project applications for this peer review cycle that were assessed in full by the Peer Review Panel (i.e. it does not include applications deemed 'Non-Competitive' after initial assessment). Mean scores (± 1 standard deviation) for each criterion are provided for each category.

Category	% of apps in Category	Criterion 1 (mean)	Criterion 2 (mean)	Criterion 3 (mean)	Criterion 4 (mean)
7	Nil	Nil	Nil	Nil	Nil
6	**6.67%**	6.333 ± 0.0	5.333 ± 0.0	5.889 ± 0.0	5.778 ± 0.0
5	**80.00%**	5.342 ± 0.443	4.435 ± 0.355	4.901 ± 0.443	5.143 ± 0.556
≤4	**13.33%**	4.068 ± 0.568	3.541 ± 0.641	3.887 ± 0.387	4.164 ± 0.564

Table 3: **Peer Reviewer Comments**

Qualitative feedback on your application is provided below in the form of Peer Reviewer comments.

**Peer Reviewer Comments:**
**Assessor Role:** Spokesperson 1 (Lead)<br>**Question:** 1. Track records of the Chief Investigators, Partner Organisations and Partner Investigators, relative to opportunity (25%)<br><br>The team is a nice mix of disciplines and different levels of experience with a track record in this area. However, it is not really clear how the consumer CI will pragmatically contribute to engagement as the consumer engagement statement does not propose practical strategies as to how consumers will guide the project. It really isn't clear what the consumer will contribute to the project as written.
**Assessor Role:** Spokesperson 1 (Lead)<br>**Question:** 2. Scientific quality of the proposal and methodology (25%)<br><br>An ambitious project in which the background could better foreground the rationale. P.1 really emphasises the ATSI problem, and then abandons it. Aboriginality emphasised in team track record and in proposal but cognisance of ATSI issues not at all evident in proposal itself. No ATSI team member. Given the emphasis on ATSI, how does the consumer engagement strategy enhance Community participation? The background is disjointed. First half in particular doesn't make clear the need for this initiative – it is just a recitation of data of what the current state of child oral health is without linking the data to statements that outline why this is important, and how it influences the proposal. It's not until we get to p.3 that it starts to make a bit of sense. Written surveys are anathema to Community – how feasible is it to expect this many Aboriginal children and their parents to complete surveys? Or other people from challenged backgrounds with poor health literacy? Risk mitigation strategy makes no mention of the very real risk of not getting proportional representation of ATSI and sociodemographically challenged groups. Aim 1 method fefers to surveys previously used (but only names one) and mentions that most surveys have been developed by the team. No indication of how valid and reliable these outcomes measures are. Aim 3 mentions team will develop their own preference-based instrument for cost effect modelling (but don't say in any detail how they will do this apart from comparing it to a 'general Aust sample'). Concern re recruitment and loss to follow up since NCOHS-2. This was barely mentioned, but could be a significant factor to consider. The applicants overplay the relationship between dental disease and outcomes at school without mentioning significant confounding effects, e.g. poverty. I am not sure that longitudinal studies or systematic reviews can resolve this question. Attributing causality is not appropriate in a longitudinal study.
**Assessor Role:** Spokesperson 1 (Lead)<br>**Question:** 3. Relevance and likelihood to influence health policy and practice (25%)<br><br>The team are proposing in partnership with government work that I would have thought was already conducted - surveillance and impact measurement of dental disease in children. Not sure this project addresses the objectives of the scheme given than the partnerships have already been created and this is part of a national program involving the Commonwealth and each State an Territory dental public health sector. Economic productivity assessment hazy and therefore its potential to influence policy and practice unclear.
**Assessor Role:** Spokesperson 1 (Lead)<br>**Question:** 4. Strength of the partnership (25%)<br><br>Partner organisations are highly appropriate implementation partners that have indicated high-levels of support. The role of the partners is not articulated in the proposal itself, this could have been better foregrounded before getting to the letters of support.
